# Radiographic Study of L5–S1 Transforaminal Endoscopic Access in a Sample from the Brazilian Population

**DOI:** 10.1055/s-0044-1793824

**Published:** 2024-12-21

**Authors:** Yoshinobu Nagasse, Caio Justino Lima, João Pedro Alves Ferreira, Edgar Takao Utino, João Paulo Bergamaschi, Helton Luís Aparecido Defino

**Affiliations:** 1 Departamento de Ortopedia e Traumatologia, Hospital Municipal Dr. Cármino Caricchio (Hospital do Tatuapé), São Paulo, SP, Brasil; 2 Especialização em Ortopedia e Traumatologia, Faculdade de Ciências Médicas da Santa Casa de São Paulo, São Paulo, SP, Brasil; 3 Curso de Especialização em Cirurgia Endoscópica da Coluna Vertebral, Faculdade de Medicina de Ribeirão Preto, Universidade de São Paulo, Ribeirão Preto, SP, Brasil; 4 Departamento de Ortopedia e Traumatologia, Faculdade de Medicina de Ribeirão Preto, Universidade de São Paulo, Ribeirão Preto, SP, Brasil

**Keywords:** discectomy, percutaneous, disc herniation, iliac crest, minimally invasive surgical procedures

## Abstract

**Objective** This study evaluated lumbar spine radiographs using the Choi and Patgaonkar classifications to verify parameters potentially influencing the L5–S1 transforaminal approach.

**Materials and Methods** We studied 167 lumbosacral spine radiographs from patients over 18 years old with no history of surgeries, tumors, fractures, or scoliosis to measure the iliac crest height and rim angle. We categorized the cases per pelvic morphology, mega-apophysis presence, and Choi and Patgaonkar classifications.

**Results** Seventy-five cases had an android pelvis and 92 had a gynecoid pelvis. The mean iliac height was 25.9 ± 7.5 cm, and the rim angle was 23.4 ± 7.5 degrees. The gynecoid pelvis showed a lower iliac height. According to Patgaonkar, 63 cases indicated a suprailiac approach, and per the Choi classification, 37 were suitable for a suprailiac approach and 106 for a suprailiac approach with foraminoplasty.

**Conclusion** Gynecoid pelvises had a lower iliac height. Furthermore, 37.7% of the cases were suitable for a suprailiac approach per the Patgaonkar classification. The Choi classification indicated a suprailiac approach for 22.1% of the cases and a suprailiac approach with foraminoplasty for 63.4% of the subjects.

## Introduction

The Kambin triangle is the base for transforaminal endoscopic surgery. 1 This anatomical structure is delimited by the superior vertebral plateau, the dural sac, and the emerging root. The space within this triangle represents a safety corridor free of noble structures. The introduction of instruments small enough to enter this space allowed the development of the transforaminal (TF) approach. 2 3 In this approach, the working cannula aligns with the intervertebral disc, crossing the safety triangle with minimal injury and joint preservation, allowing early recovery and greater joint stability. 4

However, the TF approach at the L5-S1 level is unique due to the iliac crest since the cannula introduction occurs superior to the intervertebral disc to avoid obstructions by the iliac crest, resulting in a more angled alignment about the disc, called the suprailiac (SI) approach. When the iliac crest is prominent, the SI approach may be unfeasible, requiring crest perforation, that is, a transiliac (TI) approach. Osman and Marsolais 5 validated the TI approach safety in cadaveric studies, but concerns regarding intraoperative injuries, bleeding, and pain remain.

The TF approach has benefits, as it allows foraminal decompression, avoids root manipulation, and can occur under sedation. 6 Choi et al. 7 postulated that patients whose lateral spine radiographs demonstrated an iliac crest above the lower half of the L5 pedicle tend to face significant challenges in the TF approach. Patgaonkar et al. 8 proposed a classification outlining when to select an SI or TI approach.

The present study aimed to evaluate lumbar spine radiographs using the Choi 7 and Patgaonkar et al. 8 classifications to determine the parameters potentially influencing the L5–S1 TF approach and verify the presence of mega-apophysis in the evaluated sample.

## Materials and Methods

We performed a cross-sectional study using lumbar spine radiographs from our institution, which we collected over 4 months. We included patients over 18 years old, with radiographs with adequate visualization and excluded those with previous lumbar spine surgery, tumor lesions, fractures, and scoliosis.

**Fig. 1:**
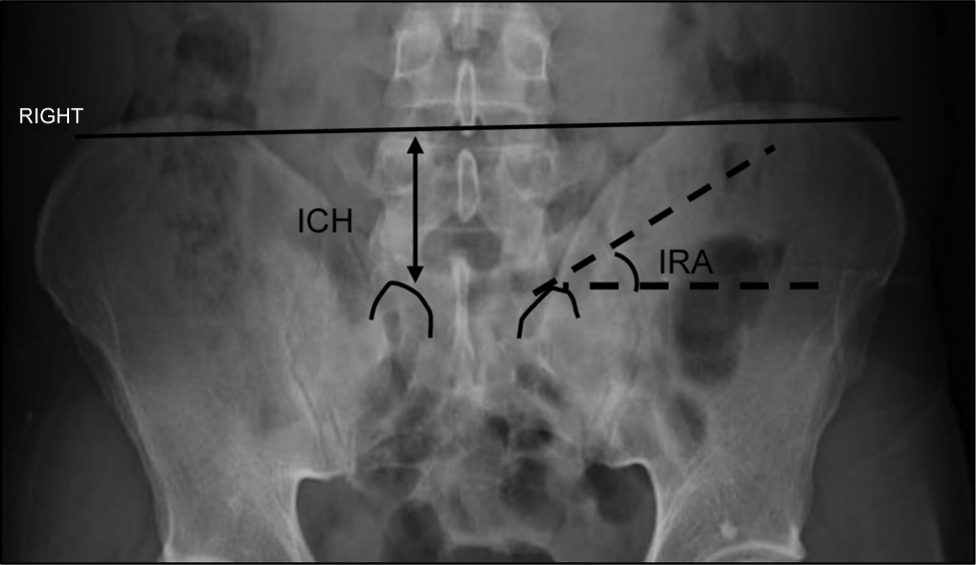
Iliac crest height (ICH; double arrow): distance between the superomedial edge of the L5–S1 vertebral joint and the line tangential to the top of the iliac crests. Iliac rim angle (IRA; dotted line): the angle between the horizontal and the line passing through the superomedial edge of the L5–S1 joint and tangential to the medial edge of the iliac crest.

We evaluated the radiographs using the Myvue system, version 11.2.2.3 (Carestream Health, Rochester, NY, USA). On the frontal radiograph, we measured the iliac crest height (ICH), that is, the vertical distance between a line tangentially connecting the tops of the iliac crests and the superomedial edge of the S1 joint, and the iliac rim angle (IRA), measured at the intersection of the horizontal line with the line passing through the superomedial edge of the S1 joint and tangentially connecting the medial edge of the iliac bone ( Fig. 1 ). The pelvis classification as android or gynecoid occurred based on its morphology, and the mega-apophysis classification followed the aspects proposed by Castellvi apud Konin and Walz. 9

**Fig. 2:**
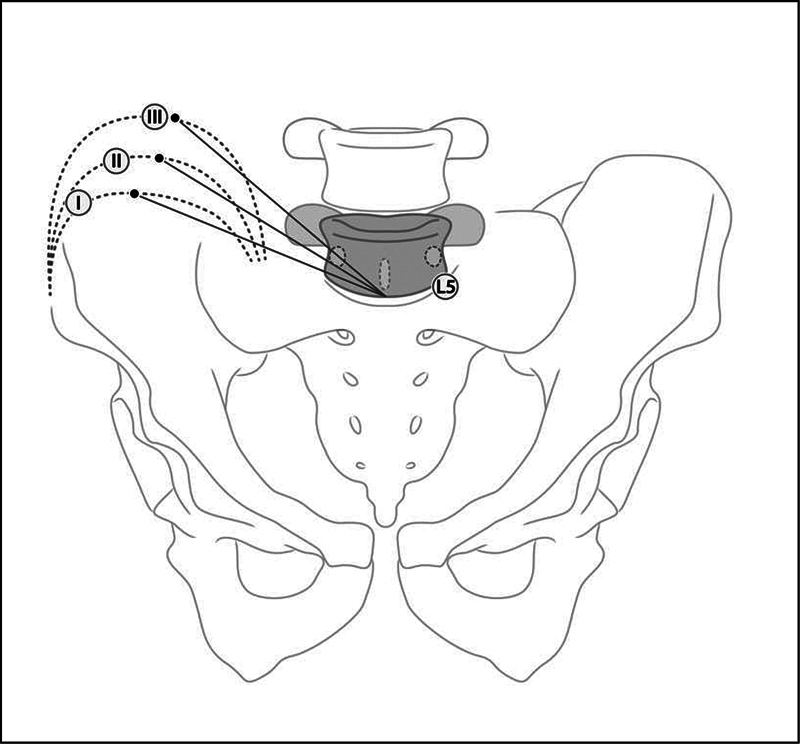
Anteroposterior Patgaonkar classification. Type I: the line between the highest point of the iliac crest and the center of the lower plateau of L5 passing below the L5 pedicle; type II: the same line passing tangentially to the lower edge of the L5 pedicle; type III: the same line passing through the L5 pedicle.

The classification by Patgaonkar et al., 8 used in anteroposterior radiographs, evaluates the relationship of the L5 pedicle with a line drawn from the top of the iliac crest to the center of the lower plateau of the L5 vertebra. In type I, the line is below the pedicle; in type II, the line passes tangent to the lower edge of the pedicle; and, in type III, the line crosses the pedicle ( Fig. 2 ). According to Patgaonkar et al., 8 patients classified as type III are suitable for a TI approach. For types I and II, the indication is an SI approach, and, for type III, a TI approach.

**Fig. 3:**
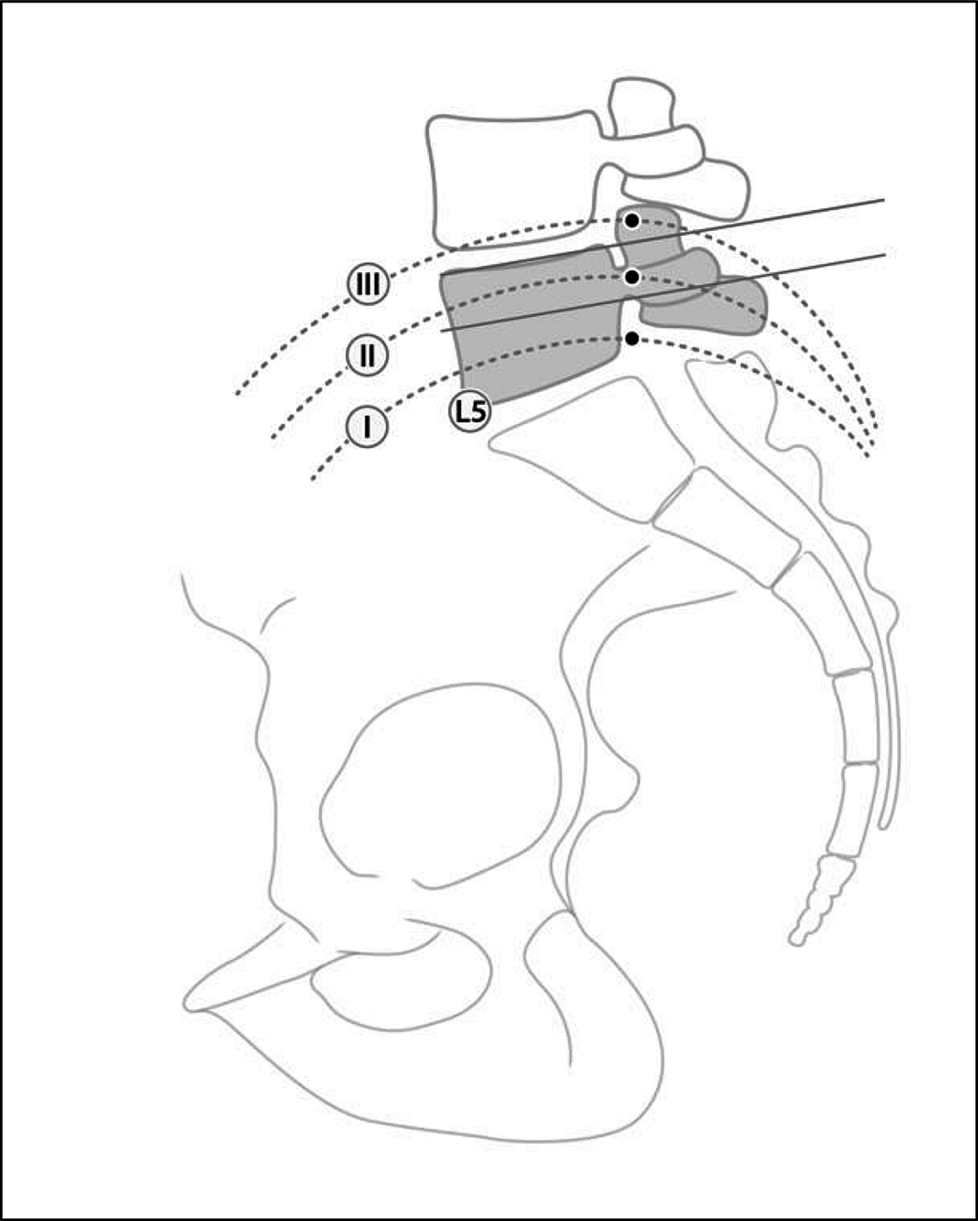
Lateral Patgaonkar classification. Type 1: the line between the highest point of the iliac crest and the center of the lower plateau of L5 passing below the pedicle of L5; type 2 the same line passing tangentially to the lower edge of the pedicle of L5; type 3: the same line passing through the pedicle of L5.

This same classification assesses lateral radiographs using the top of the iliac crest and the upper and lower edges of the L5 pedicle. In type I, the iliac crest is below the pedicle; in type II, it is at the level of the pedicle; and, in type III, it is above the pedicle ( Fig. 3 ). As in the previous classification, the author 8 considers type-III patients eligible for the TI approach. For types I and II, the indication is a single SI approach, and, for type-III cases, a TI approach.

**Fig. 4:**
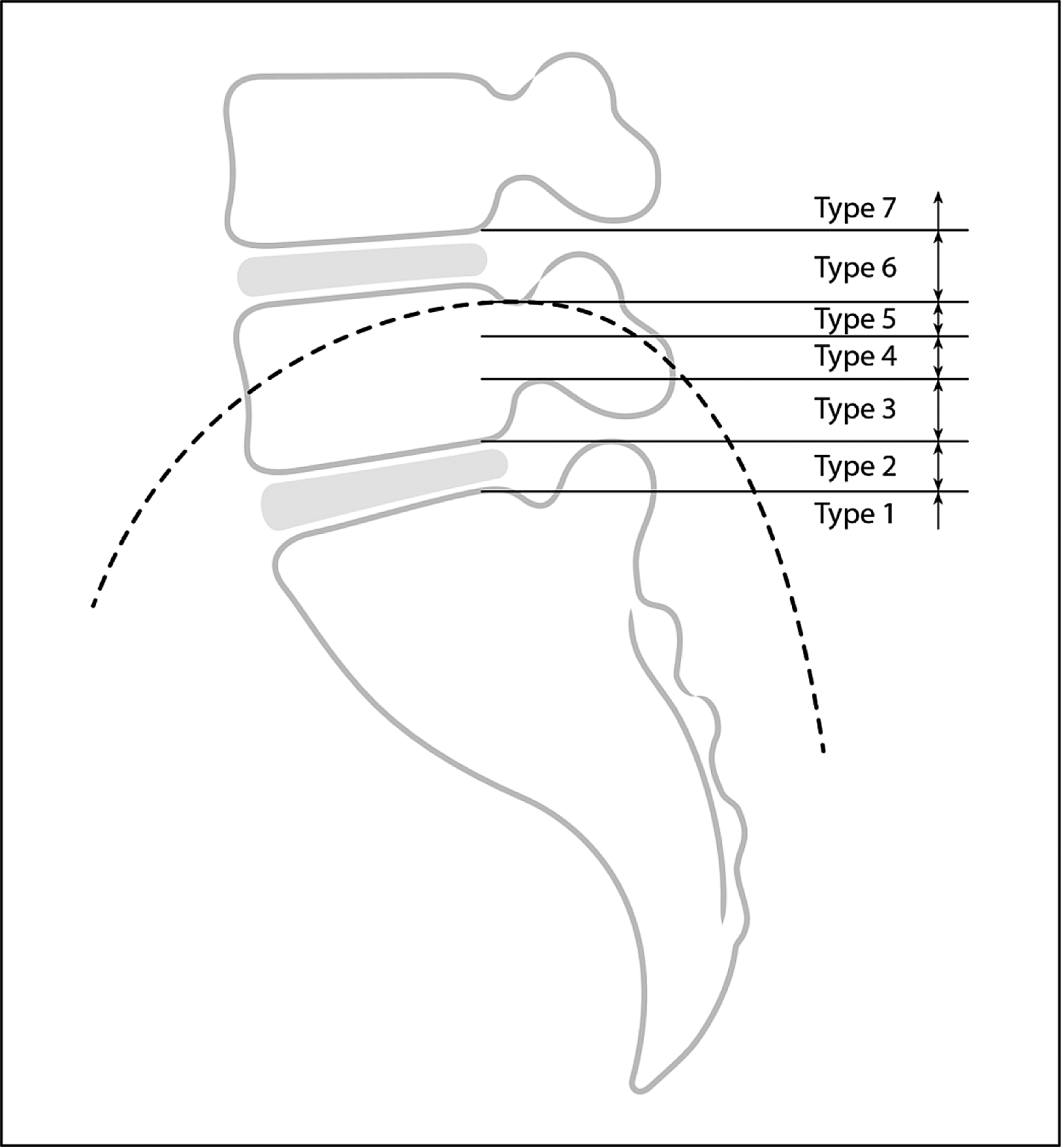
Choi classification - the diagram represents a lateral lumbar spine radiograph. Choi defined the first six reference types for the iliac crest height. We added the seventh type to include types not foreseen in the classification. The dotted curved line represents the overlap of the iliac crest on the radiograph.

Choi et al. 7 presented a similar classification, defining the height of the iliac crest by lumbar spine structures. Types 1, 2, and 3 were considered the easiest to access without the need for foraminoplasty and grouped as suitable for SI approach. In this classification, types 5 and 6 are above half of the L5 pedicle and associated with greater difficulty for the TF approach, often requiring a foraminoplasty; for these types, the indication is the TS approach with foraminoplasty. The addition of type 7 occurred only to classify iliac crests above the L4-L5 intervertebral disc, which were not foreseen by Choi et al. 7 ( Fig. 4 ).

We described the quantitative characteristics evaluated according to the approach and iliac type for the Patgaonkar classification, 8 and compared with the categories using Student's t-tests, and per the Choi classifications, 7 using analysis of variance (ANOVA). 10 We described gender and iliac type per the approaches from each classification and verified associations using likelihood ratio tests. 11

The Spearman's test calculated the correlations between ICH and IRA, illustrated as scatter diagrams. We performed the analyses in IBM SPSS Statistics for Windows, version 22.0 (IBM Corp., Armonk, NY, USA) and tabulated the data on Microsoft Excel 2013 (Microsoft Corp., Redmond, WA, USA). The significance level was 5%.

## Results

We obtained a total of 167 radiographs and described their characteristics in Table 1 . Table 2 describes their classifications according to Patgaonkar et al. 8 and Choi et al. 7

**Table 1:** Description of the classification according to Patgaonkar and Choi in all cases

Variable	Description
	(N = 167)
**Age (years)**	
Mean ± standard deviation	49.1 ± 16.7
Median (minimum–maximum)	48 (18–87)
**Gender, n (%)**	
Female	82 (49.1)
Male	85 (50.9)
**Iliac type, n (%)**	
Android	75 (44.9)
Gynecoid	92 (55.1)
**Iliac crest height (cm)**	
Mean ± standard deviation	25.9 ± 7.5
Median (minimum–maximum)	25 (6–46)
**Iliac rim angle (degrees)**	
Mean ± standard deviation	23.4 ± 7.5
Median (minimum–maximum)	22 (7–46)
**CASTELLVI, n (%)**	
0	46 (27.5)
1a	9 (5.4)
2a	11 (6.6)
3a	4 (2.4)
1b	55 (32.9)
2b	32 (19.2)
3b	10 (6)

**Table 2:** Description of the classification according to Patgaonkar and Choi in all cases

Variable: n (%)	Description
	(N = 167)
**PATGAONKAR frontal approach**	
Suprailiac	121 (72.5)
Transiliac	46 (27.5)
**PATGAONKAR lateral approach**	
Suprailiac	68 (40.7)
Transiliac	99 (59.3)
**CHOI**	
1	1 (0.6)
2	5 (3)
3	8 (4.8)
4	23 (13.8)
5	37 (22.2)
6	69 (41.3)
7	24 (14.4)
**CHOI approach**	
Suprailiac	37 (22.2)
Suprailiac with foraminoplasty	106 (63.5)
Choi classification, type 7	24 (14.4)

Table 3 shows that the TI approach per the Pantgaonkar classification in lateral radiographs was higher in males than females ( *p* < 0.001). Mean ICH and IRA were higher in patients with an indication for a TI approach per the Pantgaonkar classification ( *p* = 0.001 and *p* = 0.003, respectively), with an association between the Pantgaonkar profile and the iliac type ( *p* < 0.001). However, after adjusting for gender, age, and iliac type, the mean differences in height and angle were no longer statistically significant ( *p* > 0.05), probably due to the association of the iliac type per Pantgaonkar classification in lateral radiographs.

**Table 3:** Description of the classification according to Patgaonkar and Choi in all cases

Variable	PATGAONKAR Lateral approach		*p*	*p* *
	Suprailiac	Transiliac		
**Age (years)**			0.496**	
Mean ± standard deviation	48 ± 15.1	49.8 ± 17.7		
Median (minimum–maximum)	47 (18–84)	48 (19–87)		
**Gender, n (%)**			**< 0.001**	
Female	49 (59.8)	33 (40.2)		
Male	19 (22.4)	66 (77.6)		
**Iliac type, n (%)**			**< 0.001**	
Android	16 (21.3)	59 (78.7)		
Gynecoid	52 (56.5)	40 (43.5)		
**Iliac crest height (cm)**			0.001**	0.130
Mean ± standard deviation	23.7 ± 6.4	27.4 ± 7.9		
Median (minimum–maximum)	24 (6–36)	26 (13–46)		
**Iliac rim angle (degrees)**			0.003**	0.134
Mean ± standard deviation	21.4 ± 6.4	24.8 ± 7.9		
Median (minimum–maximum)	20.5 (7–42)	24 (11–46)		

Notes: Chi-squared test; ** Student's *t* -test; * Adjusted for age, gender, and iliac type.

Table 4 shows that men had a higher frequency of android iliac, while women had a higher frequency of gynecoid pelvis ( *p* < 0.001). Iliac crest height and IRA were higher in patients with an android pelvis ( *p* = 0.002 and *p* = 0.011, respectively). Nevertheless, after adjusting for the characteristics, only the average iliac height remained statistically higher in the android pelvis ( *p* = 0.039).

**Table 4:** Description of the iliac type according to personal characteristics and description of the quantitative features according to the approach and results of the statistical tests

Variable	Iliac type		*p*	*p* *
	Android	Gynecoid		
**Age (years)**			0.181**	
Mean ± standard deviation	47.1 ± 15.4	50.6 ± 17.6		
Median (minimum–maximum)	47 (19–81)	49 (18–87)		
**Gender, n (%)**			**< 0.001**	
Female	1 (1.2)	81 (98.8)		
Male	74 (87.1)	11 (12.9)		
**Iliac crest height (cm)**			0.002**	**0.039**
Mean ± standard deviation	27.9 ± 7.3	24.3 ± 7.3		
Median (minimum–maximum)	26 (15–46)	23 (6–46)		
**Iliac rim angle (degrees)**			0.011**	0.078
Mean ± standard deviation	25 ± 7.7	22.1 ± 7.1		
Median (minimum–maximum)	24 (11–46)	21 (7–42)		

Notes: Chi-squared test; ** Student's *t* -test; * Adjusted for age, gender, and iliac type.

Table 5 shows that the frequency of Choi classification type 7 and TS with foraminoplasty were statistically higher in men than women. Consequently, the frequency of Choi classification type 7 and TS with foraminoplasty was statistically higher in android iliac types ( *p* = 0.001). There was a mean difference in ICH and IRA between the Choi approaches when the values were not adjusted ( *p* = 0.001 and *p* = 0.004, respectively). However, after adjusting for personal features and iliac type, only ICH showed a statistically significant mean difference ( *p* = 0.042), being statistically higher for TI than SI ( *p* = 0.041) ( Table 6 ).

**Table 5:** Description of behavior per the Choi classification according to personal features and description of quantitative characteristics according to behavior and results of statistical tests

Variable	CHOI Approach			*p*	*p* *
	Suprailiac	Suprailiac with foraminoplasty	Choi et al. 7		
**Age (years)**				0.419**	
Mean ± standard deviation	46. ± 4.4	49.3 ± 17.8	52 ± 14.6		
Median (minimum–maximum)	46 (18–72)	47 (19–87)	52 (23–84)		
**Gender, n (%)**				**< 0.001**	
Female	29 (35.4)	45 (54.9)	8 (9.8)		
Male	8 (9.4)	61 (71.8)	16 (18.8)		
**Iliac type, n (%)**				**0.001**	
Android	7 (9.3)	54 (72)	14 (18.7)		
Gynecoid	30 (32.6)	52 (56.5)	10 (10.9)		
**Iliac crest height**				0.001**	**0.042**
Mean ± standard deviation	22.1 ± 6.9	26.5 ± 6.9	29.2 ± 9.1		
Median (minimum–maximum)	22 (6–36)	25.5 (13–46)	26.5 (15–46)		
**Iliac rim angle**				0.004**	0.097
Mean ± standard deviation	20.4 ± 7.3	23.7 ± 6.6	26.7 ± 9.9		
Median (minimum–maximum)	20 (7–42)	22.5 (11–43)	26 (13–46)		

**Notes:**^#^ Likelihood ratio test; **analysis of variance; *adjusted for age, gender, and iliac type; the mean iliac height was statistically higher in the transiliac than suprailiac approach ( *p* = 0.041).

**Table 6:** Comparison of the iliac crest height according to the Choi's classification

Iliac crest height					
Comparison	Mean difference	Standard error	*p*	CI (95%)	
				Inferior	Superior
Suprailiac x Suprailiac with foraminoplasty	−2.58	1.38	0.192	−5.93	0.77
Suprailiac x Choi classification, type 7	−4.73	1.89	**0.041**	−9.30	−0.15
Suprailiac with foraminoplasty x Choi classification, type 7	−2.14	1.56	0.513	−5.91	1.63

**Abbreviation:** CI, confidence interval.

**Note:** Adjusted for age, gender, and iliac type.

**Fig. 5:**
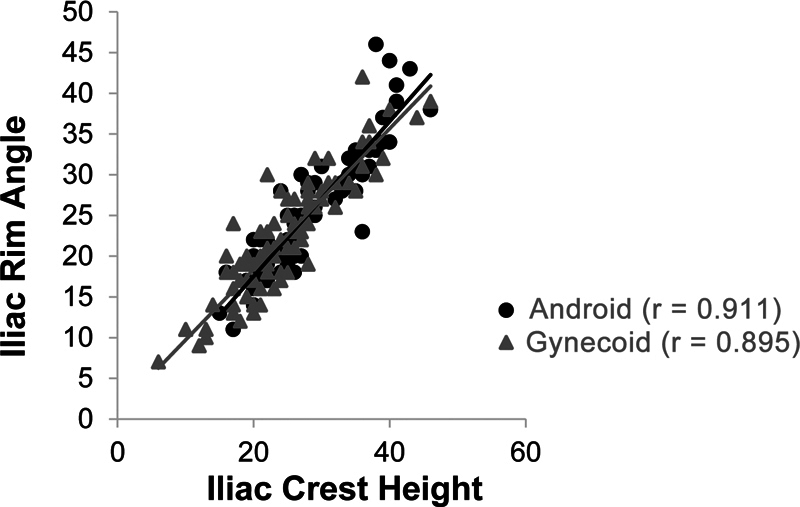
Scatter diagram of the iliac height and the iliac rim angle according to the iliac type and the result of the correlations.

Fig. 5 shows a high correlation between ICH and IRA for both iliac types (r ≈ 0.9).

## Discussion

The iliac crest is essential in the TF approach at the L5–S1 level, as it prevents disc access in alignment with its axis. This requires a foraminal approach using the SI or TI techniques. Therefore, the morphological study of the pelvis relies on three criteria, that is, ICH, IRA, and pelvic shape.

While Caldwell e Molloy apud Swelson 12 categorized the pelvic anatomy into four types—gynecoid, android, platypelloid, and anthropoid—with a focus on assessing the birth canal, our study focused specifically on the android and gynecoid variations to investigate whether the iliac shape could influence the TF approach.

We observed a predominance of the android pelvis in men (87.1%) and gynecoid pelvis in women (98.8%). In addition to a predominance of gynecoid pelvis, females presented a smaller average ICH (24.3 ± 7.3 cm) than males (27.9 ± 7.3 cm), consistent with another study demonstrating that women tend to have a lower iliac crest than men. 13 The IRA had no difference in any of the variables studied.

The classification of Patgaonkar et al. 8 in lateral radiographs assesses the height of the iliac crest about the L5 vertebra. Both the shape of the pelvis and the gender of the patient demonstrated a significant impact on the choice of the approach. However, after statistical adjustments considering gender and pelvic type, the difference in ICH and IRA becomes insignificant. The fact that female patients had a smaller ICG may have influenced the results before adjustment.

In summary, the Patgaonkar et al. 8 classification recommended the SI approach in 63 cases (37.7%) and the TI approach in 104 cases (62.3%). Of the latter, only five cases had an indication for the TI approach based solely on the anteroposterior radiograph. Another 41 cases were suitable for the TI approach in both radiographic views, while 58 were suitable based on the lateral radiographs alone.

The Choi et al. 7 classification, which evaluates the height of the iliac crest and the L5 vertebra on lateral radiographs, received an addition, type 7, to encompass cases in which the ICH exceeds the estimates of the original classification of Choi et al. 7 As in Patgaonkar et al. 8 classification, we observed significant variations related to gender and pelvic shape.

After adjusting for gender and iliac type, we found that the difference in ICH remained significant. The group with indication for an SI approach had a mean ICH of 22.1 ± 6.9 cm, significantly lower than the mean value for the type-7 group (29.2 ± 9.1 cm).

In addition, we evaluated the correlation between ICH and IRA through a scatter plot. We detected a strong correlation (r ≈ 0.9) between the 2 metrics, indicating that an increased ICH corresponds to increased IRA values. Interestingly, the numerical values for ICH and IRA tend to be close to each other.

Finally, 65.8% of the cases analyzed did not present mega-apophyses or showed only type 1a or 1b mega-apophyses patterns according to the Castellvi classification. These patterns have a minimal impact on the TF approach.

## Conclusion

The elevated iliac crest is associated with higher grades in the Choi classification. The IRA, although well correlated with ICH, did not show the same statistical difference between the classifications.
