# Supplementary material for: Radiographic Study of L5–S1 Transforaminal Endoscopic Access in a Sample from the Brazilian Population
Source: Rev Bras Ortop (Sao Paulo). 2024 Dec 21;59(6):e847–53. doi: 10.1055/s-0044-1793824 (PMC11663076; doi:10.1055/s-0044-1793824)
Supplement: Related texts

# Research article: Estudo radiográfico do acesso endoscópico transforaminal L5-S1 em uma amostra da população brasileira

**Keywords:** crista ilíaca, discectomia percutânea, hérnia de disco, procedimentos cirúrgicos minimamente invasivos

## Abstract

**Objetivo** Avaliar radiografias da coluna lombar utilizando as classificações de Choi e Patgaonkar, e verificar parâmetros que possam influenciar o acesso transforaminal L5–S1.

**Métodos** Cento e sessenta e sete radiografias de coluna lombossacra de pacientes maiores de 18 anos, sem históricos de cirurgias, tumores, fraturas ou escoliose, foram estudadas. Aferiu-se a altura da crista ilíaca e o ângulo da borda do ilíaco. Os casos foram categorizados conforme morfologia pélvica, presença de megapófise e classificações de Choi e de Patgaonkar.

**Resultados** Setenta e cinco casos exibiram pelve androide, e 92 ginecoide. A média da altura do ilíaco foi de 25,9 ± 7,5 cm, e do ângulo de borda do ilíaco foi de 23,4 ± 7,5 graus. A pelve ginecoide mostrou menor altura do ilíaco. Segundo a classificação de Patgaonkar, 63 casos indicaram acesso suprailíaco; pela classificação de Choi, 37 foram considerados aptos para acesso suprailíaco e 106 para acesso suprailíaco com foraminoplastia.

**Conclusão** Pelves ginecoides tiveram menor altura do ilíaco. Além disso, 37,7% dos casos foram aptos para acesso suprailíaco pela classificação de Patgaonkar. De acordo com a classificação de Choi, 22,1% estavam aptos para o acesso suprailíaco e 63,4% foram considerados aptos para acesso suprailíaco com foraminoplastia.

## Introdução

A cirurgia endoscópica transforaminal é baseada no triângulo de Kambin. 1 Esta estrutura anatômica é delimitada pelo platô superior da vértebra, pelo saco dural e pela raiz emergente. O espaço dentro deste triângulo representa um corredor de segurança livre de estruturas nobres. O surgimento de instrumentais compactos o suficiente para se adaptarem a este espaço permitiram o desenvolvimento do acesso transforaminal (TF). 2 3 Nesta abordagem, a cânula de trabalho fica alinhada com o disco intervertebral e atravessa o triângulo de segurança com mínima lesão e preservação da articulação, permitindo assim uma recuperação precoce e maior preservação da estabilidade articular. 4

Entretanto, a abordagem transforaminal (TF) no nível L5–S1 é única devido à crista ilíaca, pois a cânula é introduzida superiormente ao disco intervertebral para evitar obstruções pelo ilíaco, resultando em alinhamento mais angulado em relação ao disco, denominado acesso suprailíaco (SI). Quando a crista ilíaca é proeminente, o acesso SI pode ser inviável, requerendo perfuração da crista, originando o acesso transilíaco (TI). Osman e Marsolais 5 validaram a segurança do acesso TI em estudos com cadáveres, mas há preocupações quanto a lesões, sangramentos e dor intraoperatórios.

O acesso TF apresenta benefícios, pois permite a descompressão foraminal, evita manipulação das raízes e é possível realizá-la sob sedação. 6 Choi et al. 7 postulou que pacientes cujas radiografias da coluna em perfil demonstram uma crista ilíaca situada acima da metade inferior do pedículo de L5 tendem a enfrentar desafios significativos no acesso TF. Por sua vez, Patgaonkar et al. 8 propôs uma classificação delineando quando é apropriado optar por uma abordagem SI ou TI.

O objetivo deste trabalho foi avaliar radiografias de coluna lombar através das classificações de Choi 7 e de Patgaonkar et al., 8 aferir parâmetros que possam influenciar no acesso transforaminal L5–S1, e verificar a presença de megapófise na amostra avaliada.

## Materiais e Métodos

Foi realizado um estudo transversal com radiografias da coluna lombar realizadas em nossa instituição, onde foram coletadas radiografias de coluna lombossacra, realizadas no período de 4 meses. Foram incluídos pacientes com idade maior que 18 anos, com radiografia com visualização adequada, e excluídos aqueles com presença de cirurgia de coluna lombar prévia, lesões tumorais, fraturas e escoliose.

As radiografias foram avaliadas pelo sistema Myvue, versão 11.2.2.3 (Carestream Health, Rochester, NY, Estados Unidos). Na radiografia frontal foram medidas a altura da crista ilíaca (ACI), representada pela distância vertical entre a linha que tangencia os topos das cristas ilíacas e a borda superomedial da articulação de S1; e o ângulo da borda do ilíaco (ABI), aferido no cruzamento da linha horizontal com a linha que passa pela borda súpero medial da articulação de S1 e tangencia a borda medial do ilíaco ( Fig. 1 ). A bacia foi classificada como androide ou ginecoide conforme sua morfologia, e foi realizada classificação de megapófise conforme proposto por Castellvi apud Konin e Walz. 9

**Fig. 1:** Altura de crista ilíaca (seta dupla): distância entre borda superomedial da articulação L5–-S1 vertebral e a linha que tangencia o topo das cristas ilíacas. Ângulo de borda de ilíaco (linha pontilhada): ângulo entre horizontal e linha que passa pela borda superomedial da articulação L5–S1 e tangencia a borda medial da crista ilíaca.

A classificação de Patgaonkar et al., 8 aplicada na radiografia anteroposterior, avalia a relação do pedículo de L5 com uma linha traçada do topo da crista ilíaca ao centro do platô inferior da vértebra de L5. No tipo I, a linha está abaixo do pedículo, no tipo II, a linha passa tangente à borda inferior do pedículo, e no tipo III, a linha cruza o pedículo ( Fig. 2 ). Conforme Patgaonkar et al., 8 pacientes classificados como tipo III têm indicação de abordagem TI. Os tipos I e II foram agrupados como abordagem SI, e o tipo III como abordagem TI.

**Fig. 2:** Classificação Patgaonkar em radiografia anteroposterior Tipo I: a linha entre o ponto mais alto da crista ilíaca e o centro do platô inferior de L5 passa abaixo do pedículo de L5; tipo II: a mesma linha passa tangenciando a borda inferior do pedículo de L5; tipo III: a mesma linha passa pelo pedículo de L5.

Nesta mesma classificação há uma avaliação na radiografia de perfil, que usa o topo da crista ilíaca e a borda superior e inferior do pedículo de L5. No tipo I, a crista ilíaca está abaixo do pedículo, no tipo II, está na altura do pedículo, e no tipo III, está acima do pedículo ( Fig. 3 ). Assim como na classificação prévia, o autor 8 considera pacientes do tipo III elegíveis para a abordagem TI. Foram considerados o tipo I e II como um grupo único de abordagem SI, e os casos tipo III como abordagem TI.

**Fig. 3:** Classificação Patgaonkar em radiografia de perfil. Tipo 1: a linha entre o ponto mais alto da crista ilíaca e o centro do platô inferior de L5 passa abaixo do pedículo de L5; tipo 2: a mesma linha passa tangenciando a borda inferior do pedículo de L5; tipo 3: a mesma linha passa pelo pedículo de L5.

Choi et al. 7 apresentaram uma classificação semelhante, na qual a altura da crista ilíaca é definida por estruturas da coluna lombar. Os tipos 1, 2 e 3 foram considerados os de maior facilidade de acesso, sem necessidade de foraminoplastia, e esses tipos foram agrupados como indicados para abordagem SI. Nesta classificação, os tipos 5 e 6 estão acima da metade do pedículo de L5 e são associados a maior dificuldade no acesso TF, muitas vezes necessitando de foraminoplastia para o acesso, sendo estes dois tipos agrupados como SI com foraminoplastia. Foi acrescentado um tipo 7 apenas para classificar cristas ilíacas acima do disco intervertebral L4–L5, que não foram previstos no estudo de Choi et al. 7 ( Fig. 4 ).

**Fig. 4:** Classificação de Choi - o esquema representa uma radiografia de coluna lombar em perfil. Os 6 primeiros tipos de referência para altura de crista ilíaca foram definidas por Choi. O sétimo tipo foi acrescentado para incluir tipos não previsto para a classificação. A linha curva pontilhada representaria a sobreposição da crista ilíaca na radiografia.

As características quantitativas avaliadas foram descritas segundo conduta e tipo de ilíaco para as incidências da escala Patgaonkar, 8 e comparadas entre as categorias com o uso de testes *t* de Student, e segundo as categorias da escala Choi, 7 com uso de análise de variância ( *analysis of variance* , ANOVA, em inglês), 10 enquanto o sexo e tipo de ilíaco foram descritos segundo as condutas de cada escala, e a associação foi verificada com o uso de testes da razão de verossimilhanças. 11

Correlações entre ACI e ABI foram calculadas através da correlação Spearman e ilustradas através do diagrama de dispersão. As análises foram feitas no IBM SPSS Statistics for Windows, versão 22.0 (IBM Corp., Armonk, NY, Estados Unidos) e os dados tabulados com Microsoft Excel 2013 (Microsoft Corp., Redmond, WA, Estados Unidos). Os testes foram realizados com nível de significância de 5%.

## Resultados

Foram obtidas 167 radiografias cujas características foram descritas na Tabela 1 . Na Tabela 2 estão descritas suas classificações conforme Patgaonkar et al. 8 e Choi et al. 7

**Tabela 1:** Descrição das características avaliadas em todos os pacientes

Variável	Descrição
	(N = 167)
**Idade (anos)**	
média ± desvio padrão	49,1 ± 16,7
mediana (mínimo–máximo)	48 (18–87)
**Sexo, n (%)**	
Feminino	82 (49,1)
Masculino	85 (50,9)
**Tipo de Ilíaco, n (%)**	
Androide	75 (44,9)
Ginecoide	92 (55,1)
**Altura da crista ilíaca (cm)**	
média ± desvio padrão	25,9 ± 7,5
mediana (mínimo–máximo)	25 (6–46)
**Ângulo de borda do ilíaco (graus)**	
média ± desvio padrão	23,4 ± 7,5
mediana (mínimo–máximo)	22 (7–46)
**CASTELLVI, n (%)**	
0	46 (27,5)
1a	9 (5,4)
2a	11 (6,6)
3a	4 (2,4)
1b	55 (32,9)
2b	32 (19,2)
3b	10 (6)

**Tabela 2:** Descrição da Classificação conforme Patgaonkar e Choi em todos os casos

Variável: n (%)	Descrição
	(N = 167)
**Conduta PATGAONKAR frente**	
Suprailíaco	121 (72,5)
Transilíaco	46 (27,5)
**Conduta PATGAONKAR perfil**	
Suprailíaco	68 (40,7)
Transilíaco	99 (59,3)
**CHOI**	
1	1 (0,6)
2	5 (3)
3	8 (4,8)
4	23 (13,8)
5	37 (22,2)
6	69 (41,3)
7	24 (14,4)
**Conduta CHOI**	
Suprailíaco	37 (22,2)
Suprailíaco com foraminoplastia	106 (63,5)
Classificação 7 de Choi	24 (14,4)

Na Tabela 3 , a abordagem TI pela escala de Pantgaonkar em radiografias de perfil foi maior nos homens do que nas mulheres ( *p* < 0,001). A ACI e o ABI foram, em média, maiores nos pacientes com abordagem de Pantgaonkar em radiografias de perfil e abordagem TI ( *p* = 0,001 e *p* = 0,003, respectivamente), havendo associação entre a Pantgaonkar em radiografias de perfil e o tipo de ilíaco ( *p* < 0,001). Porém, após ajustados o sexo, idade e tipo de ilíaco, as diferenças médias na altura e ângulo não continuaram mais estatisticamente significativas ( *p* > 0,05), provavelmente pela associação do tipo de ilíaco com a Pantgaonkar em radiografias de perfil.

**Tabela 3:** Descrição da conduta pela escala Patgaonkar perfil segundo as características pessoais e descrição das características quantitativas segundo conduta e resultado dos testes estatísticos

Variável	Conduta PATGAONKAR perfil		*p*	*p* *
	Suprailíaco	Transilíaco		
**Idade (anos)**			0,496**	
média ± desvio padrão	48 ± 15,1	49,8 ± 17,7		
mediana (mínimo–máximo)	47 (18–84)	48 (19–87)		
**Sexo, n (%)**			**< 0,001**	
Feminino	49 (59,8)	33 (40,2)		
Masculino	19 (22,4)	66 (77,6)		
**Tipo de Ilíaco, n (%)**			**< 0,001**	
Androide	16 (21,3)	59 (78,7)		
Ginecoide	52 (56,5)	40 (43,5)		
**Altura da crista ilíaca (cm)**			0,001**	0,130
média ± desvio padrão	23,7 ± 6,4	27,4 ± 7,9		
mediana (mínimo–máximo)	24 (6–36)	26 (13–46)		
**Ângulo de borda do ilíaco (graus)**			0,003**	0,134
média ± desvio padrão	21,4 ± 6,4	24,8 ± 7,9		
mediana (mínimo–máximo)	20,5 (7–42)	24 (11–46)		

**Notas:** Teste do qui-quadrado; **teste *t* de Student; *ajustado pela idade, sexo e tipo de ilíaco.

A Tabela 4 mostra que homens apresentaram maior frequência de ilíaco androide, enquanto mulheres apresentaram maior frequência de pelve ginecoide ( *p* < 0,001). A ACI e o ABI foram maiores em pacientes com ilíaco androide ( *p* = 0,002 e *p* = 0,011, respectivamente), mas após ajuste das características, apenas a ACI permaneceu em média estatisticamente maior nos ilíacos androides ( *p* = 0,039).

**Tabela 4:** Descrição do tipo de ilíaco segundo as características pessoais e descrição das características quantitativas segundo conduta e resultado dos testes estatísticos

Variável	Tipo de Ilíaco		*p*	*p* *
	Androide	Ginecoide		
**Idade (anos)**			0,181**	
média ± desvio padrão	47,1 ± 15,4	50,6 ± 17,6		
mediana (mínimo–máximo)	47 (19–81)	49 (18–87)		
**Sexo, n (%)**			**< 0,001**	
Feminino	1 (1,2)	81 (98,8)		
Masculino	74 (87,1)	11 (12,9)		
**Altura da crista ilíaca (cm)**			0,002**	**0,039**
média ± desvio padrão	27,9 ± 7,3	24,3 ± 7,3		
mediana (mínimo–máximo)	26 (15–46)	23 (6–46)		
**Ângulo de borda do ilíaco (graus)**			0,011**	0,078
média ± desvio padrão	25 ± 7,7	22,1 ± 7,1		
mediana (mínimo–máximo)	24 (11–46)	21 (7–42)		

**Notas:** Teste do qui-quadrado; **teste *t* de Student; *ajustado pela idade e sexo.

A Tabela 5 mostra que, nos homens, a frequência da classificação 7 de Choi e suprailíaco com foraminoplastia foram estatisticamente maiores nos homens que nas mulheres. Consequentemente, a frequência da classificação 7 de Choi e suprailíaco com foraminoplastia foi estatisticamente maior nos tipos de ilíaco androide ( *p* = 0,001). Houve diferença média na ACI e no ABI entre as condutas pelo Choi, quando não ajustados os valores ( *p* = 0,001 e *p* = 0,004, respectivamente). Porém, após ajuste das características pessoais e do tipo de ilíaco, apenas a ACI apresentou diferença média estatisticamente significativa ( *p* = 0,042), sendo estatisticamente maior no TI que no SI ( *p* = 0,041), como demonstrado na Tabela 6 .

**Tabela 5:** Descrição da conduta pela escala Choi segundo as características pessoais e descrição das características quantitativas segundo conduta e resultado dos testes estatísticos

Variável	Conduta CHOI			*p*	*p* *
	Suprailíaco	Suprailíaco com foraminoplastia	Choi et al. 7		
**Idade (anos)**				0,419**	
média ± desvio padrão	46,3 ± 14,4	49,3 ± 17,8	52 ± 14,6		
mediana (mínimo–máximo)	46 (18–72)	47 (19–87)	52 (23–84)		
**Sexo, n (%)**				**< 0,001**	
Feminino	29 (35,4)	45 (54,9)	8 (9,8)		
Masculino	8 (9,4)	61 (71,8)	16 (18,8)		
**Tipo de Ilíaco, n (%)**				**0,001**	
Androide	7 (9,3)	54 (72)	14 (18,7)		
Ginecoide	30 (32,6)	52 (56,5)	10 (10,9)		
**Altura da crista ilíaca**				0,001**	**0,042**
média ± desvio padrão	22,1 ± 6,9	26,5 ± 6,9	29,2 ± 9,1		
mediana (mínimo–máximo)	22 (6–36)	25,5 (13–46)	26,5 (15–46)		
**Ângulo de borda do ilíaco**				0,004**	0,097
média ± desvio padrão	20,4 ± 7,3	23,7 ± 6,6	26,7 ± 9,9		
mediana (mínimo–máximo)	20 (7–42)	22,5 (11–43)	26 (13–46)		

**Notas:**^#^ Teste da razão de verossimilhanças; **análise de variância; *ajustado pela idade, sexo e tipo de ilíaco; A altura do ilíaco foi em média estatisticamente maior no transilíaco que suprailíaco ( *p* = 0,041).

**Tabela 6:** Comparação da altura de crista ilíaca conforme classificação de conduta de Choi

Altura de crista ilíaca					
Comparação	Diferença média	Erro padrão	*p*	IC (95%)	
				Inferior	Superior
Suprailíaco x Suprailíaco com foraminoplastia	−2,58	1,38	0,192	−5,93	0,77
Suprailíaco x Classificação 7 de Choi	−4,73	1,89	**0,041**	−9,30	−0,15
Suprailíaco com foraminoplastia x Classificação 7 de Choi	−2,14	1,56	0,513	−5,91	1,63

**Abreviatura:** IC, intervalo de confiança.

**Nota:** Ajustado pela idade, sexo e tipo de ilíaco.

A Fig. 5 mostra que a correlação entre a ACI e o ABI é bastante alta para ambos os tipos de ilíacos (r ≈ 0,9).

**Fig. 5:** Diagrama de dispersão entre a altura do ilíaco e o ângulo de borda do ilíaco segundo tipo de ilíaco e resultado das correlações.

## Discussão

A crista ilíaca é fundamental na abordagem TF no nível L5–S1, pois impede o acesso ao disco alinhado ao seu eixo. Isso exige que a abordagem do forame seja realizada com acesso SI ou TI. Por isso, foi feito o estudo morfológico da pelve baseado em três critérios: ACI, ABI e formato de bacia.

Enquanto Caldwell e Molloy apud Swelson 12 categorizaram a anatomia pélvica em quatro tipos—ginecoide, androide, platipeloide e antropoide—com foco na avaliação do canal de parto, o presente estudo centrou-se especificamente nas variações androide e ginecoide, pois objetivo foi investigar se o formato do ilíaco poderia influenciar no acesso TF.

Dessa forma, foi observado predomínio de pelve androide em homens (87,1%), e pelve ginecoide nas mulheres (98,8%). Os casos de pacientes do sexo feminino, além de apresentarem predominância de pelve ginecoide, também apresentaram a ACI em média (24,3 ± 7,3 cm) menor que casos de pacientes do sexo masculino (27,9 ± 7,3 cm), confirmando o achado observado em outro estudo cujo resultado demonstrou que mulheres tendem a ter uma crista ilíaca mais baixa que homens. 13 O ABI não apresentou diferença em nenhuma das variáveis pesquisadas.

A classificação de Patgaonkar et al. 8 nas radiografias em perfil avalia a ACI em relação à vértebra L5. Tanto o formato da pelve quanto o sexo do paciente demonstraram impacto significativo na escolha do método de acesso. No entanto, quando ajustes estatísticos são feitos levando em conta o sexo e o tipo de pelve, a diferença em relação à ACI e ao ABI torna-se insignificante. O fato de pacientes do sexo feminino estarem associados a menores ACI pode ter influenciado o resultado antes do ajuste.

Em suma, a classificação de Patgaonkar et al. 8 recomendou o acesso SI em 63 casos (37,7%), e o acesso TI em 104 casos (62,3%). Destes últimos, apenas 5 casos receberam indicação para a abordagem TI com base unicamente na radiografia anteroposterior. Outros 41 casos receberam indicação para acesso TI em ambas as vistas radiográficas, enquanto 58 casos foram indicados com base exclusiva na radiografia lateral.

À classificação de Choi et al., 7 que avalia a ACI e a vértebra L5 com base nas radiografias em perfil, foi acrescentado um tipo 7 para abranger casos quando a ACI supera as estimativas da classificação original de Choi et al. 7 Tal qual na classificação de Patgaonkar, 8 observamos variações significativas relacionadas ao sexo e ao formato da pelve.

Após ajustar para sexo e tipo ilíaco, constatamos que a diferença no ACI permaneceu significativa. O grupo com indicação de abordagem SI exibiu uma média de ACI de 22,1 ± 6,9 cm, que foi significativamente menor que a média observada no grupo tipo 7 (29,2 ± 9,1 cm).

Além disso, avaliamos a correlação entre a ACI e o ABI por meio de um diagrama de dispersão. Foi observada uma forte correlação (r ≈ 0,9) entre as duas métricas, indicando que um aumento nos valores do ABI está associado a um aumento correspondente nos valores da ACI. Curiosamente, os valores numéricos para ABI e ACI tendem a ser próximos entre si.

Por último, 65,8% dos casos analisados não apresentaram megapófises, ou mostraram apenas padrões tipo 1a ou 1b de megapófises de acordo com a classificação de Castellvi, padrões estes que têm impacto mínimo no acesso TF.

## Conclusão

A crista ilíaca elevada está associada a graduações mais altas da classificação de Choi. O ABI, apesar de ter boa correlação com a ACI, não apresentou a mesma diferença estatística entre as classificações.
